# Prefabricated Skin Excision in Face Lift: A Simplified Technique

**DOI:** 10.29252/wjps.8.1.62

**Published:** 2019-01

**Authors:** Abdoljalil Kalantar-Hormozi, Soraya Shahrokh, Ali Abbaszadeh-kasbi, Nazanin Rita Davai

**Affiliations:** 1 Department of Plastic and Craniofacial Surgery, 15 Khordad Hospital, Medical College, Shahid Beheshti University of Medical Sciences, Tehran, Iran;; 2 Department of Plastic and Craniofacial Surgery, Modarres Hospital, Medical College, Shahid Beheshti University of Medical Sciences, Tehran, Iran;; 3 School of Medicine, Tehran University of Medical Sciences, Tehran, Iran;; 4 Private Practice, Tehran, Iran

**Keywords:** Face lift, Rhytidectomy, Facial cosmetic surgery

## Abstract

**BACKGROUND:**

The demand for facial plastic surgery has dramatically been increased in recent years. Over the last decade, numerous methods have been improved for facelift surgery. Despite these modifications, skin excision technique has not changed significantly. In this study, authors have tried to introduce a new technique regarding skin excision at the initial step of facelift surgery.

**METHODS:**

A prospective study from 2012 to 2017 on 52 patients was carried out to apply a new technique for facelift ‘’Prefabricated skin excision method’’ for all eligible patients undergoing facelift surgery. The skin calling for excision was marked by the surgeon, and then, an analgesic drug was administered. Then, excision of the marked part of the skin was performed and afterward the dissection of the superficial musculoaponeurotic system (SMAS) was performed with the direct exposure.

**RESULTS:**

All patients were female, and 50 (96.1%) cases were primary face lift and 2 (3.9%) cases were secondary. There were no complications among the patients.

**CONCLUSION:**

Facilitating the manipulation of deep layer, using this technique led to the further exposure of the surgical site, and more preferable hemostasis was achieved as well.

## INTRODUCTION

Aging is a progressive process to a great extent unpreventable at least at the present time, occurring with the time.^1^^-^^3^ Not only surgical methods, but non-surgical methods have also been evolved to counteract the effects of aging on the face.^4^^-^^7^ Among these methods, face lift surgery, clinically known as rhytidectomy, is the procedure of choice used to rejuvenate the appearance of the face and jaw by reducing the appearance of wrinkles and other signs of aging. Facelift surgery has evolved in parallel with our more advanced understanding of the anatomy of facial aging.^8^^-^^10^ Hence, the demand for facial plastic surgery has dramatically been increased in recent years as more people from all socioeconomic levels and age groups have become interested in facial rejuvenation. Meanwhile, As the population ages, the demand for esthetic surgery in the elderly is increasing at a greater rate.^11^^,^^12^

For over a century, innovative surgeons have developed a wide variety of methods to rejuvenate age-related changes, especially in the face.^8^ To date, although it seems that developed face lift methods have more differences than similarities, they have far more in common.^13^ Almost all face lift methods have addressed the various depths of dissection, but few methods have dealt with skin management of face lift.^9^^,^^10^^,^^14^^-^^17^ The Aim of this study was an introduction of a new method for skin excision at the first stage of face lift surgery to improve the exposure and hemostasis, and reducing complications as well as increasing direct vision on deep plan procedures. 

## MATERIALS AND METHODS

A prospective study from 2012 to 2017 was conducted for all patients undergoing our new technique for facelift of prefabricated skin excision method. Informed consent was obtained from all individual participants included in the study. Overall 52 patients were enrolled. Those patients who did not cease the smoking 4 weeks prior the surgery; those who were not able to discontinue the anticoagulants, antiplatelet agents, or nonsteroidal anti-inflammatory drugs therapy based on cardiologist or hematologist consultants; and those who had uncontrolled hypertension were excluded from the study, because they were considered as poor candidate for facelift surgery. All surgeries were performed by the senior author in the private practice. Patients were visited by the senior author after one week, one, 6 and 12 months of the surgery.

For all patients, a throughout medical history, as well as physical examination, were performed by the senior author. Routine laboratory evaluations such as complete blood count (CBC) with differential, PT, PTT, INR, EKG, chest X-ray, sodium, potassium, glucose, and urine analysis were requested. All cases also had a psychological consultation to evaluate and exclude any major disorder that precludes any aesthetic operation, such as obsessive compulsive disease (OCD), body dysmorphic disease (BDD), etc. The complementary laboratory tests were tailored to the types of specific issues. For women in childbearing ages, pregnancy was excluded through urine analysis, unless an effective contraceptive method was being used by the patient or his/her spouse. Moreover, an inquiry was made regarding smoking and using herbal drugs such as chondroitin, ephedra, echinacea, glucosamine, ginkgo biloba, goldenseal, milk thistle, ginseng, kava, fish oil, licorice, red chili pepper, feverfew, and garlic.^18^^-^^20^

All patients were asked to discontinue the anticoagulants, antiplatelet agents, nonsteroidal anti-inflammatory, vitamin E, and herbal drugs therapy 2 weeks before the surgery.^10^^,^^19^^-^^21^ With existing a serious medical illness, the decision about the surgery was made in conjunction with a cardiologist or a hematologist. Smoking was not permitted for at least 4 weeks before the surgery; a urine toxicology test was considered for smoker patients immediately before the surgery.^10^^,^^21^^-^^23^

**Fig. 1:**
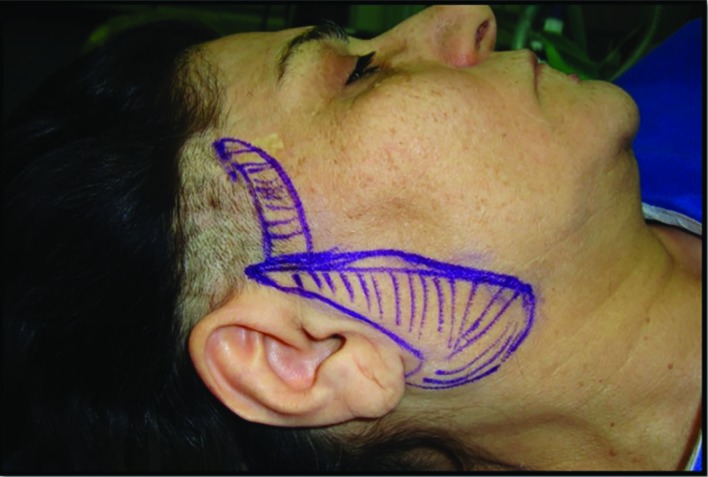
Preoperational marking for pre-hair line incision, and excision of prefabricated skin

**Fig. 2:**
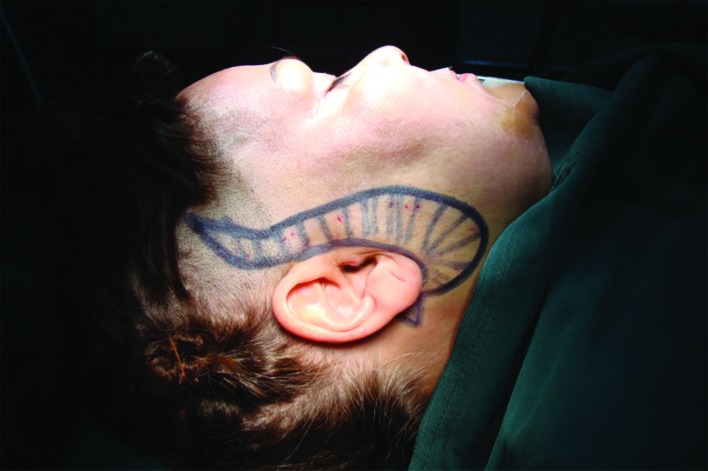
Preoperational marking for intra-hair line incision, and excision of prefabricated skin

All procedures performed with intravenous sedation combined with local anesthesia after prep and drape and at the first step marking was performed (Figures 1 and 2). Our preferred method for incision was preauricular incision that could be extended through the hairline or behind it based on patient’s condition. Consequently, we estimated the amount of skin which should be removed with pinch test. Anterior border of the margin that must be excised had been marked. This anterior marking must be the mirror image of the initial preauricular incision.

At this time, the tumescent injection was performed. Twenty-five milliliters of 2% lidocaine with 25 ml of normal saline/1/100000 epinephrine was injected to each side of the face. Ten minutes after the injection, the procedure was initiated with the excision of the prefabricated skin from the area that had been marked (Figure 1 and 2). Then dissection of malar skin and subcutaneous flap performed under the direct vision. Dissection was continued until the nasolabial fold in the face and a region preferred by the surgeon in the neck. The next steps could be varying according to a method preferred by a surgeon who can do any of the facelift methods such as superficial musculoaponeurotic system (SMAS) plication, SMAS excision, or deep plane technique. Our preferred method was SMAS plication.

**Fig. 3:**
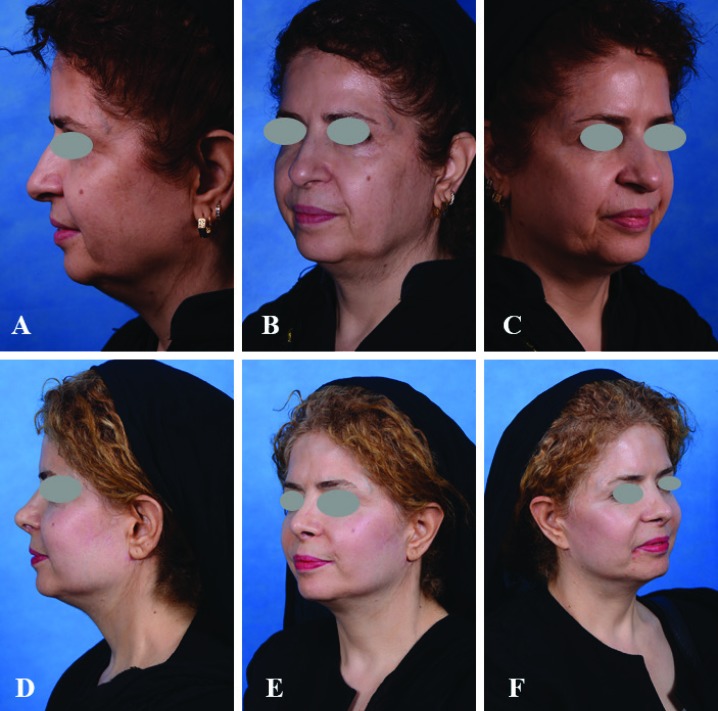
A, B, C) A 56-year-old woman before the face lift surgery. D, E, F) The same patient 12 months after prefabricated skin excision face lift

After the completion of hemostasis, the wound was closed in subcutaneous and skin layers with ethicon-monocryl® (poliglecaprone 25- 3.0 and 4.0) suture. We did not use any drain for any of patients. Only after completion of one side, injection of the other side was initiated and then the same procedure was repeated. Only a light dressing for the first 24 hours was used and then was removed. We did not use any anti-inflammatory drugs, ice pack, or steroids. Photographs were taken 6 months after the surgery and later (Figure 3).

## RESULTS

The mean age of patients was 52.1±3.1 years, ranging from 41 to 63 years. Fifty patients (96.1%) were primary cases while 2 (3.9%) cases were secondary, one which means had another facelift surgery previously. All patients were female. Preauricular incision was used for all patients, and in 30 (57.7%), it was intra-hairline, while it was hairline in 22 (42.3%) patients. All patients underwent SMAS plication. The average time of the surgery for each side of the face was around 90 minutes, and for the whole face was approximately 180 minutes. All patients were discharged from the surgery center, the next morning with oral analgesic and antibiotic for 3 days. There was not any complication in the patients, including hematoma, nerve damage, or infection. The scar of surgery was acceptable.

## DISCUSSION

Aging is a process in which nearly all organs of the body are affected, unfavorable for elderly people. Among the entire affected organs, the appearance of the face is notably affected by aging, and up to now, what is recognized in process of facial aging is that changes in skin, soft tissue, bone, and fat tissue play a prominent role in facial aging while most changes are result in great part from changes in the skin.^9^^,^^24^^-^^27^ Such various procedures as either invasive or non-invasive have been developed to rejuvenate the facial appearance, having various efficacies and costs.^9^^,^^10^^,^^28^^,^^29^

Because the facelift, today, is known as the most effective aesthetic procedure among cosmetic surgeons in rejuvenating and reshaping the face, more people, especially women than men, are seeking face lifting surgery. During the last years, increasing demand for rejuvenating technique resulted notably in the improvement in both surgical and non-surgical rejuvenating techniques.^9^^-^^11^^,^^30^^,^^31^ The first case of surgical treatment of rhytids, consisted of a small number of strips of skin excised, was operated in 1912 in Berlin by a German plastic surgeon.^32^

In 1920 and 1921, two surgeons, independently, described the first method, Skin-only method, of subcutaneous rhytidectomy consisted of extensive undermining and lipectomy. Some major benefits of the technique are simplicity, rapid postoperative recovery time, and minimized risk for the facial nerve injury while some main drawbacks of the method are minimal effect on underlying facial shape, distortion of the face due to the excess tension on skin flap.^8^^,^^12^^,^^17^^,^^33^^,^^34^ Introducing post-tragal incision, aiming at improving the earlobe appearance and decreasing in earlobe malposition in 1928 was a significant contribution.^17^^,^^35^ After 46 years, the first technique of modern facelift, subfacial dissection was introduced, and afterward the SMAS was introduced in 1976.^36^

Since then many techniques have been described for facelift such as deep subcutaneous facelift (including robust and thick flap and no risk of facial nerve injury as for benefits), subcutaneous facelift with SMAS manipulation with suture or SMAS plication (including low complication rates and less recovery time as for benefits in comparison to more aggressive SMAS flap procedures), lateral SMASectomy or subcutaneous facelift with SMAS removal (which has an increased risk of injury to facial nerve branches in anteriorly extended dissections), extended and high SMAS flaps (have been developed to rejuvenate the midface; although facial nerve branches are at risk, can be easily avoided with using tumescent infiltration), deep plane or subcutaneous facelift with separate SMAS flap (there is the same risk of facial nerve injury as in the other methods including sub-SMAS dissection), composite (making malar fat repositioning possible unlike deep plane technique), and subperiosteal facelift (facial nerve is not endangered; the method has quite little effect on facial skin).^9^^,^^10^^,^^14^^-^^16^^,^^37^^-^^42^

Despite these differences among these techniques, none of them is superior to the others.^43^^-^^45^ In spite of different methods, skin management in facelift plays an important role in the final result. Because in almost all techniques, excess skin excision is the last step of procedure it may make difficult for achieving perfect exposure and hemostasis, it makes more trouble for surgery in deep plan and also proceeds complications. We attempted to introduce our new technique of facelift named prefabricated skin excision, with the aim of facilitating procedure and consequently increasing the safety of the operation. When the skin is excised before the SMAS dissection, a dissection with good exposure and hemostasis substantially minimizing the risk of facial nerve injury can be performed. There was no case of neural damage, hematoma, or skin necrosis among our patients during the study. The only disadvantage of this new method is that the high learning curve is needed. Our new method focusing at managing skin at the beginning of the facelift surgery increases the safety of facelift surgery and facilitate it. 

## References

[1] Harman D (1981). The aging process. Proceedings of the National Academy of Sciences.

[2] Salminen A, Kaarniranta K, Kauppinen A (2013). Crosstalk between Oxidative Stress and SIRT1: Impact on the Aging Process. Int J Mol Sci.

[3] Bratic A, Larsson NG (2013). The role of mitochondria in aging. J Clin Invest.

[4] Carruthers JA, Lowe NJ, Menter MA, Gibson J, Nordquist M, Mordaunt J, Walker P, Eadie N, Group BGLIS (2002). A multicenter, double-blind, randomized, placebo-controlled study of the efficacy and safety of botulinum toxin type A in the treatment of glabellar lines. J Am Acad Dermatol.

[5] Johl SS, Burgett RA (2006). Dermal filler agents: a practical review. Curr Opin Ophthalmol.

[6] Baker TJ (1962). Chemical face peeling and rhytidectomy. A combined approach for facial rejuvenation. Plast Reconstr Surg Transplant Bull.

[7] Negishi K, Wakamatsu S, Kushikata N, Tezuka Y, Kotani Y, Shiba K (2002). Full-face photorejuvenation of photodamaged skin by intense pulsed light with integrated contact cooling: initial experiences in Asian patients. Lasers Surg Med.

[8] Rohrich RJ, Pessa JE (2007). The fat compartments of the face: anatomy and clinical implications for cosmetic surgery. Plast Reconstr Surg.

[9] Warren RJ, Aston SJ, Mendelson BC (2011). Face lift. Plast Reconstr Surg.

[10] Derby BM, Codner MA (2017). Evidence-Based Medicine: Face Lift. Plast Reconstr Surg.

[11] American Society of Plastic Surgeons [Internet] (2014 ). Cosmetic Plastic Surgery Statistics: Cosmetic Procedure Trends.

[12] Adamson PA, Litner JA (2005). Evolution of rhytidectomy techniques. Facial Plast Surg Clin North Am.

[13] Lambros V, Stuzin JM (2008). The cross-cheek depression: surgical cause and effect in the development of the “joker line” and its treatment. Plast Reconstr Surg.

[14] Hoefflin SM (1998). The extended supraplatysmal plane (ESP) face lift. Plast Reconstr Surg.

[15] Robbins LB, Brothers DB, Marshall DM (1995). Anterior SMAS plication for the treatment of prominent nasomandibular folds and restoration of normal cheek contour. Plast Reconstr Surg.

[16] Stuzin JM, Baker TJ, Gordon HL, Baker TM (1995). Extended SMAS dissection as an approach to midface rejuvenation. Clin Plast Surg.

[17] DeFatta RJ, Williams EF (2009). Evolution of midface rejuvenation. Arch Facial Plast Surg.

[18] Heller J, Gabbay JS, Ghadjar K, Jourabchi M, O’hara C, Heller M, Bradley JP (2006). Top-10 list of herbal and supplemental medicines used by cosmetic patients: what the plastic surgeon needs to know. Plast Reconstr Surg.

[19] Wong WW, Gabriel A, Maxwell GP, Gupta SC (2012). Bleeding risks of herbal, homeopathic, and dietary supplements: a hidden nightmare for plastic surgeons?. Aesthet Surg J.

[20] Chin SH, Cristofaro J, Aston SJ (2009). Perioperative management of antidepressants and herbal medications in elective plastic surgery. Plast Reconstr Surg.

[21] Guyuron B (2010). An evidence-based approach to face lift. Plast Reconstr Surg.

[22] Chang LD, Buncke G, Slezak S, Buncke HJ (1996). Cigarette smoking, plastic surgery, and microsurgery. J Reconstr Microsurg.

[23] Rees TD, Liverett DM, Guy CL (1984). The effect of cigarette smoking on skin-flap survival in the face lift patient. Plast Reconstr Surg.

[24] Kligman LH (1986). Photoaging Manifestations, prevention, and treatment. Dermatol Clin.

[25] Jelks GW, Jelks EB (1991). The influence of orbital and eyelid anatomy on the palpebral aperture. Clin Plast Surg.

[26] Pessa JE, Chen Y (2002). Curve analysis of the aging orbital aperture. Plast Reconstr Surg.

[27] Bartlett SP, Grossman R, Whitaker LA (1992). Age-related changes of the craniofacial skeleton: an anthropometric and histologic analysis. Plast Reconstr Surg.

[28] Rigotti G, Charles-de-Sa L, Gontijo-de-Amorim NF, Takiya CM, Amable PR, Borojevic R, Benati D, Bernardi P, Sbarbati A (2016). Expanded Stem Cells, Stromal-Vascular Fraction, and Platelet-Rich Plasma Enriched Fat: Comparing Results of Different Facial Rejuvenation Approaches in a Clinical Trial. Aesthet Surg J.

[29] Qureshi AA, Parikh RP, Sharma K, Myckatyn TM, Tenenbaum MM (2017). Nonsurgical Facial Rejuvenation: Outcomes and Safety of Neuromodulator and Soft-Tissue Filler Procedures Performed in a Resident Cosmetic Clinic. Aesthetic Plast Surg.

[30] Baker DC, Aston SJ, Guy CL, Rees TD (1977). The male rhytidectomy. Plast Reconstr Surg.

[31] Goisis M, Di Petrillo A, Rinna C, Brillante C, Guareschi M, Youssef DA (2014). Fillers in Aesthetic Medicine Injections in Aesthetic Medicine.

[32] Hollander F, Joseph M (1912). Cosmetic surgery. Handbuch der Kosmetik.

[33] Bettman AG (1920). Plastic and cosmetic surgery of the face. North West Med.

[34] Bourguet J ( 1921). La chirurgie esthétique de la face. Concours Med.

[35] Joseph J (1931). Nasenplastik und sonstige Gesichtsplastik nebst einem Anhangu¨ ber Mammaplastik und einige weitere Operationen aus dem Geseite dera¨ usseren Ko¨ rperplastik: ein Atlas und Lehrbuch.

[36] Mitz V, Peyronie M (1976). The superficial musculo-aponeurotic system (SMAS) in the parotid and cheek area. Plast Reconstr Surg.

[37] Berry MG, Davies D (2010). Platysma-SMAS plication facelift. J Plast Reconstr Aesthet Surg.

[38] Marten TJ (2008). High SMAS facelift: combined single flap lifting of the jawline, cheek, and midface. Clin Plast Surg.

[39] Owsley JQ Jr (1977). Platysma-fascial rhytidectomy: a preliminary report. Plast Reconstr Surg.

[40] Hamra ST (1992). Repositioning the orbicularis oculi muscle in the composite rhytidectomy. Plast Reconstr Surg.

[41] Hamra ST (1992). Composite rhytidectomy. Plast Reconstr Surg.

[42] Tessier P (1989). [Subperiosteal face-lift]. Ann Chir Plast Esthet.

[43] Sinno S, Schwitzer J, Anzai L, Thorne CH (2015). Face-Lift Satisfaction Using the FACE-Q. Plast Reconstr Surg.

[44] Chang S, Pusic A, Rohrich RJ (2011). A systematic review of comparison of efficacy and complication rates among face-lift techniques. Plast Reconstr Surg.

[45] Lambros V (2008). Models of facial aging and implications for treatment. Clin Plast Surg.

